# Predisposing factors for admission to intensive care units of patients with COVID-19 infection—Results of the German nationwide inpatient sample

**DOI:** 10.3389/fpubh.2023.1113793

**Published:** 2023-02-15

**Authors:** Karsten Keller, Ioannis T. Farmakis, Luca Valerio, Sebastian Koelmel, Johannes Wild, Stefano Barco, Frank P. Schmidt, Christine Espinola-Klein, Stavros Konstantinides, Thomas Münzel, Ingo Sagoschen, Lukas Hobohm

**Affiliations:** ^1^Department of Cardiology, University Medical Center of the Johannes Gutenberg-University Mainz, Mainz, Germany; ^2^Center for Thrombosis and Hemostasis (CTH), University Medical Center of the Johannes Gutenberg-University Mainz, Mainz, Germany; ^3^Medical Clinic VII, Department of Sports Medicine, University Hospital Heidelberg, Heidelberg, Germany; ^4^Department of Internal Medicine, Triemli Hospital Zurich, Zurich, Switzerland; ^5^Department of Angiology, University Hospital Zurich, Zurich, Switzerland; ^6^Department of Cardiology, Mutterhaus Trier, Trier, Germany; ^7^Department of Cardiology, Democritus University of Thrace, Alexandroupolis, Greece; ^8^German Center for Cardiovascular Research (DZHK), Partner Site Rhine Main, Mainz, Germany

**Keywords:** COVID-19, SARS-CoV-2, healthcare resources, intensive care unit (ICU), mortality, case-fatality

## Abstract

**Background:**

Intensive care units (ICU) capacities are one of the most critical determinants in health-care management of the COVID-19 pandemic. Therefore, we aimed to analyze the ICU-admission and case-fatality rate as well as characteristics and outcomes of patient admitted to ICU in order to identify predictors and associated conditions for worsening and case-fatality in this critical ill patient-group.

**Methods:**

We used the German nationwide inpatient sample to analyze all hospitalized patients with confirmed COVID-19 diagnosis in Germany between January and December 2020. All hospitalized patients with confirmed COVID-19 infection during the year 2020 were included in the present study and were stratified according ICU-admission.

**Results:**

Overall, 176,137 hospitalizations of patients with COVID-19-infection (52.3% males; 53.6% aged ≥70 years) were reported in Germany during 2020. Among them, 27,053 (15.4%) were treated in ICU. COVID-19-patients treated on ICU were younger [70.0 (interquartile range (IQR) 59.0–79.0) vs. 72.0 (IQR 55.0–82.0) years, *P* < 0.001], more often males (66.3 vs. 48.8%, *P* < 0.001), had more frequently cardiovascular diseases (CVD) and cardiovascular risk-factors with increased in-hospital case-fatality (38.4 vs. 14.2%, *P* < 0.001). ICU-admission was independently associated with in-hospital death [OR 5.49 (95% CI 5.30–5.68), *P* < 0.001]. Male sex [OR 1.96 (95% CI 1.90–2.01), *P* < 0.001], obesity [OR 2.20 (95% CI 2.10–2.31), *P* < 0.001], diabetes mellitus [OR 1.48 (95% CI 1.44–1.53), *P* < 0.001], atrial fibrillation/flutter [OR 1.57 (95% CI 1.51–1.62), *P* < 0.001], and heart failure [OR 1.72 (95% CI 1.66–1.78), *P* < 0.001] were independently associated with ICU-admission.

**Conclusion:**

During 2020, 15.4% of the hospitalized COVID-19-patients were treated on ICUs with high case-fatality. Male sex, CVD and cardiovascular risk-factors were independent risk-factors for ICU admission.

## Introduction

During December 2019 first pneumonia cases of unknown origin were detected in China. The causative pathogen was identified as severe acute respiratory syndrome coronavirus 2 (SARS-CoV-2) (1, 2). Patients with SARS-CoV-2 infections, also shortly named as coronavirus disease 2019 (COVID-19), presented in in- and outpatient settings (1, 3). First COVID-19 cases in Germany were detected at the end of January 2020 in Bavaria (3, 4) and a strong and fast spread from this initial cluster in the German population was observed (3, 5). This spread of SARS-CoV-2 infections was accompanied by a previously unprecedented strain on healthcare systems worldwide (6).

In the first wave of the disease in 2020, the German healthcare system had the advantage that several European countries faced this strain some weeks before. Thus, German hospitals were in part able to benefit from experiences made in other healthcare systems in terms of risk stratification for outpatient care, hospital and ICU admissions. Nevertheless, in the early phase of the COVID-19-pandemic, decisions for risk stratification and ICU admission of COVID-19-patients were primarily based on physicians' experience regarding health care management of critical care and the unsorted reports of colleagues all over the world in the light of pending study results.

In previously published studies analyzing also the German nationwide inpatient sample, we have shown that the in-hospital case-fatality rate of hospitalized patients with confirmed COVID-19-infection was ~18% in Germany during the year 2020 (3, 7) and the case-fatality rate increased dramatically if treatment on intensive care units (ICU) and/or mechanical ventilation were needed (3, 7–9). Since a large number of patients with severe respiratory and cardiovascular complications of COVID-19-infection had to be treated on ICU, in some areas, ICUs were completely overloaded (3, 6, 7, 10, 11). Thus, ICU has to be considered as a bottleneck regarding health care planning and threatening critical overload of the national healthcare systems (3, 6–11). ICU availability, admission policy and health care structure vary across Europe as additionally the demographics and government-policies do (3, 6).

Beside the previously published results, it is of outstanding interest to understand determinants of ICU admission and outcome, which are both crucial factors for adequate health care planning, decision making and pandemic management (3, 6). Therefore, we aimed to analyze the ICU admission and case-fatality rate in Germany and to identify characteristics and outcomes of patient admitted to ICU in order to identify predictors and associated conditions for worsening and case-fatality for this critical ill patients' group.

## Methods

### Data source

The Research Data Center (RDC) of the Federal Bureau of Statistics (Wiesbaden, Germany) calculated the statistical analyses and provided aggregated statistic-results on the basis of our SPSS codes (IBM Corp. Released 2011. IBM SPSS Statistics for Windows, Version 20.0. IBM Corp: Armonk, NY, USA), which were previously supplied by us to the RDC (source: RDC of the Federal Statistical Office and the Statistical Offices of the federal states, DRG Statistics 2020, own calculations) (12, 13).

**Figure 1:**
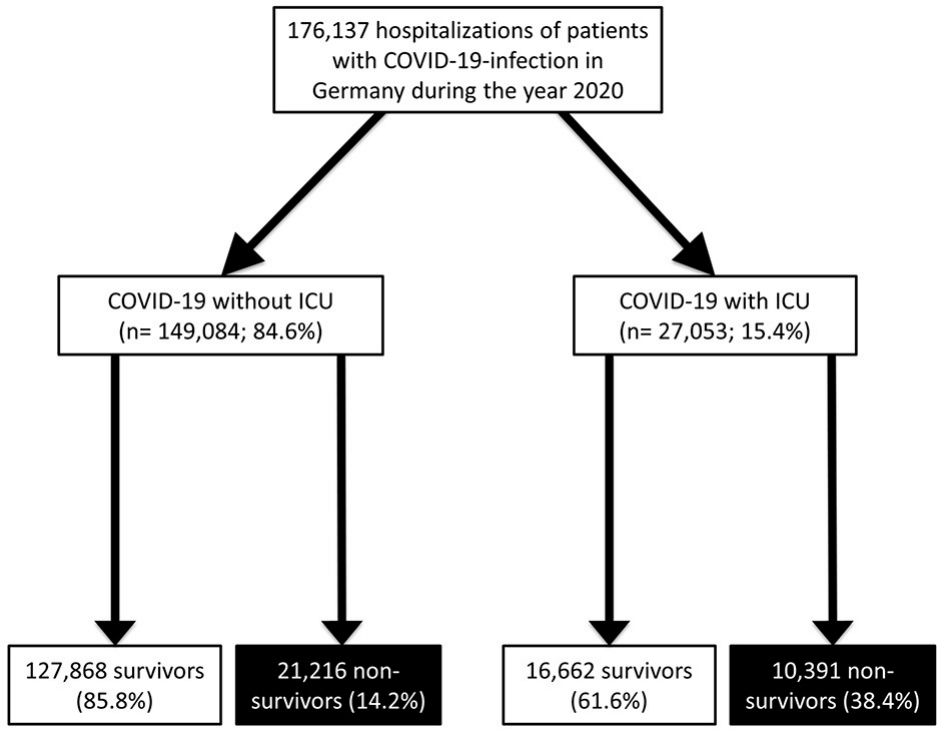
Flow-chart. COVID-19, coronavirus disease 2019; ICU, intensive care unit.

With this computed analysis of the German nationwide inpatient sample, we aimed to analyze temporal trends of all hospitalized patients with a confirmed COVID-19 diagnosis. All patients, who were treated in German hospitals with a COVID-19 infection confirmed by a laboratory test (ICD-code U07.1) during the observational period between January 1st and December 31st of the year 2020 were included in the present study. The COVID-19 patients were stratified for ICU treatment and we identified independent predictors of ICU admission during hospitalization (Figure 1).

### Study oversight and support

Since in the present study the investigators did not accessed directly individual patient data but only summarized results provided by the RDC, approval by an ethics committee as well as patients' informed consent were not required, in accordance with German law (12, 13).

### Coding of diagnoses, procedures, and definitions

After introduction of a diagnosis- and procedure-related remuneration system in Germany in the year 2004, coding according the German Diagnosis Related Groups (G-DRG) system with coding of patient data on diagnoses, coexisting conditions, and on surgeries/procedures/interventions and transferring these data/codes to the Institute for the Hospital Remuneration System is mandatory for German hospitals to get their remuneration (10, 11). Therefore, patients' diagnoses are coded according to the International Statistical Classification of Diseases and Related Health Problems, 10th revision, with German modification (ICD-10-GM) (10, 11). In addition, surgical/diagnostic/interventional procedures are coded according to OPS-codes (Operationen- und Prozedurenschlüssel). In our present of the German nationwide inpatient sample, we were able to identify all hospitalized patients with a confirmed COVID-19 diagnosis (ICD-code U07.1) in Germany during the year 2020 (COVID-19 as main or secondary diagnosis).

Post-COVID-19 was defined as a status of previous survived COVID-19-infection before the patient's hospitalization with the actual COVID-19 infection.

### Study outcomes

Primary study endpoint was admission on ICU. In addition, we analyzed occurrence of all-cause in-hospital death and the prevalence of major adverse cardiovascular and cerebrovascular events [MACCE, composite of all-cause in-hospital death, acute myocardial infarction (ICD-code I21), and/or ischemic stroke (ICD-code I63)].

Furthermore, we analyzed the occurrence of the aggravated respiratory manifestations pneumonia (ICD codes J12-J18) and acute respiratory distress syndrome (ARDS, ICD code J80) as well as other adverse events during hospitalization such as cardio-pulmonary resuscitation (OPS-code 8-77), venous thromboembolism (ICD codes I26, I80, I81, and I82), acute kidney failure (ICD-code N17), myocarditis (ICD code I40), myocardial infarction (acute and recurrent, ICD codes I21 and I22), stroke (ischemic or hemorrhagic, ICD codes I61-I64), intracerebral bleeding (ICD code I61), gastro-intestinal bleeding (ICD code K92.0, K92.1, and K92.2) and transfusion of blood constituents (OPS code 8-800). The outcomes were defined according current guidelines (14–23). The acute respiratory distress syndrome (ARDS) was defined in 1994 by the American-European Consensus Conference (AECC) and revised in 2011 with the Berlin Definition (15).

### Statistical analysis

Differences in patient characteristics between the groups of hospitalized COVID-19-patients with ICU treatment vs. without ICU treatment were calculated with Wilcoxon-Whitney *U*-test for continuous variables and Fisher's exact or chi^2^-test for categorical variables, as appropriate. Temporal trends regarding hospitalizations of COVID-19-patients with ICU treatment and in-hospital mortality over time and as well as trend-changes with increasing age were estimated by means of linear regression analyses. Results were presented as β-estimates and 95% confidence intervals (CI). Logistic regression models were calculated to investigate associations between (I) patients' characteristics and ICU-admission as well as (II) associations between adverse events and ICU-admission. Furthermore, we calculated logistic regression models to analyse (III) the associations of patients' characteristics and in-hospital death in ICU-patients as well as (IV) the associations of adverse events during in-hospital course and in-hospital death in ICU-patients. In order to warrant that the results of the mentioned logistic regressions are not substantially biased by other influencing factors and therefore, guarantying a widely independence of important different cofactors during hospitalization, the multivariable logistic regressions were adjusted for age, sex, diabetes mellitus, cancer, heart failure, coronary artery disease, chronic obstructive pulmonary disease, essential arterial hypertension, chronic renal insufficiency (glomerular filtration rate <60 ml/min/1,73 m^2^), atrial fibrillation/flutter, hyperlipidemia, and obesity.

Results were presented as Odds Ratios (OR) and 95% CI. All statistical analyses were carried out with the use of SPSS software (IBM Corp. Released 2011. IBM SPSS Statistics for Windows, Version 20.0. IBM Corp: Armonk, NY, USA). Only two-sided *P*-values <0.05 were considered to be statistically significant. No adjustment for multiple testing was applied.

## Results

### Baseline characteristics

During the year 2020, 176,137 hospitalizations (52.3% males; 53.6% aged 70 years or older) of patients with confirmed COVID-19-infection were reported in German hospitals. Of these inpatients, 27,053 (15.4%) were admitted to ICU, while overall, 31,607 (17.9%) died during hospitalization (Figure 1). ICU admission in COVID-19 patients was associated with increased case-fatality [univariate regression: OR 3.76 (95% CI 3.65–3.87), *P* < 0.001; multivariate regression: OR 5.49 (95% CI 5.30–5.68), *P* < 0.001].

**Figure 2:**
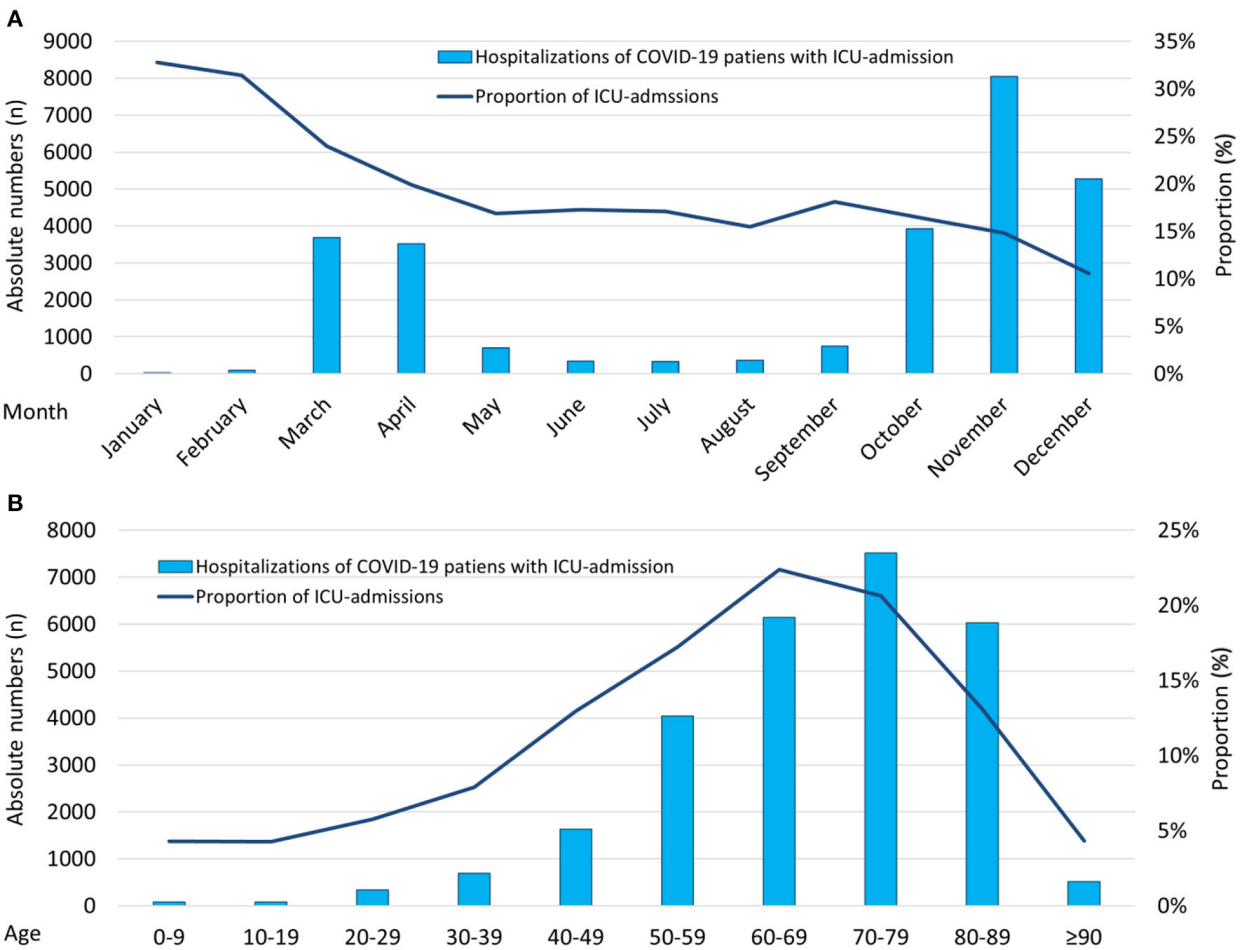
Temporal trends regarding total numbers of patients with COVID-19-infection admitted to ICU. **(A)** Temporal trends regarding total numbers of hospitalized patients with COVID-19-infection admitted to ICU (absolute numbers: blue bars; relative numbers: blue line) stratified for months. **(B)** Temporal trends regarding total numbers of hospitalized patients with COVID-19-infection admitted to ICU (absolute numbers: blue bars; relative numbers: blue line) stratified for age decades. COVID-19, coronavirus disease 2019; ICU, intensive care unit.

The monthly percentage of COVID-19 patients admitted to ICUs of German hospitals decreased over time from 32.8% in January 2020 to a minimum of 14.8% in November 2020 [β −0.89 (95% CI −0.93 to −0.85), *P* < 0.001], while highest absolute numbers of total ICU admissions were observed in spring and winter of the year 2020 (Figure 2A). ICU admissions related to all COVID-19 hospitalizations increased with inclining age [β 0.11 (95% CI 0.09–0.14), *P* < 0.001] with a maximum in the 8th decade of life (27.8%; Figure 2B).

### Comparison of COVID-19-patients admitted to ICU vs. those without ICU treatment

As aforementioned, ~15% of the hospitalized patients with COVID-19 in Germany were admitted to ICUs who were in median 2 years younger [70.0 (Interquartile range (IQR) 59.0–79.0) vs. 72.0 (IQR 55.0–82.0) years, *P* < 0.001] and more often of male sex (66.3 vs. 48.8%, *P* < 0.001) compared to those hospitalized, but treated outside the ICU (Table 1). COVID-19 patients admitted to ICU had more frequently cardiovascular risk factors (CVRF) and cardiovascular diseases (CVD) as well as lung and kidney diseases than those without ICU-treatment resulting in higher Charlson comorbidity index in ICU treated patients [5.0 (IQR 3.0–7.0) vs. 4.0 (IQR 1.0–6.0), *P* < 0.001; Table 1]. As expected, the aggravated respiratory manifestations of COVID-19-infections such as pneumonia (89.2 vs. 55.5%, *P* < 0.001) and acute respiratory distress syndrome (ARDS, 35.4 vs. 1.4%, *P* < 0.001) were more frequently found in patients, who were in need of intensive care treatment (Table 1).

**Table 1:** Patients' characteristics, medical history, presentation, and adverse in-hospital events of the 176,137 hospitalized patients with confirmed COVID-19 infection in Germany in the year 2020 stratified for ICU treatment.

**Parameters**	**COVID-19 without ICU (*n* = 149,084; 84.6%)**	**COVID-19 with ICU (*n* = 27,053; 15.4%)**	***P*-value**
Age	72.0 (55.0 / 82.0)	70.0 (59.0 / 79.0)	**<0.001**
Age ≥70 years	80,277 (53.8%)	14,052 (51.9%)	**<0.001**
Female sex	74,834 (50.2%)	9,115 (33.7%)	**<0.001**
In-hospital stay (days)	7.0 (3.0 / 12.0)	16.0 (9.0 / 26.0)	**<0.001**
**Cardiovascular risk factors**			
Obesity	6,557 (4.4%)	2,826 (10.4%)	**<0.001**
Diabetes mellitus	35,581 (23.9%)	9,651 (35.7%)	**<0.001**
Essential arterial hypertension	68,080 (45.7%)	14,400 (53.2%)	**<0.001**
Hyperlipidemia	22,651 (15.2%)	4,922 (18.2%)	**<0.001**
**Comorbidities**			
Coronary artery disease	20,174 (13.5%)	5,400 (20.0%)	**<0.001**
Heart failure	20,521 (13.8%)	6,598 (24.4%)	**<0.001**
Peripheral artery disease	4,398 (3.0%)	1,242 (4.6%)	**<0.001**
Atrial fibrillation/flutter	26,478 (17.8%)	7,682 (28.4%)	**<0.001**
Chronic obstructive pulmonary disease	9,486 (6.4%)	2,668 (9.9%)	**<0.001**
Chronic renal insufficiency (glomerular filtration rate <60 ml/min/1,73 m^2^)	22,494 (15.1%)	4,878 (18.0%)	**<0.001**
Cancer	7,416 (5.0%)	1,585 (5.9%)	**<0.001**
Vasculopathy	218 (0.1%)	112 (0.4%)	**<0.001**
Charlson comorbidity index	4.0 (1.0 / 6.0)	5.0 (3.0 / 7.0)	**<0.001**
**Respiratory manifestations of COVID-19**			
Pneumonia	82,784 (55.5%)	24,129 (89.2%)	**<0.001**
Acute respiratory distress syndrome	2,025 (1.4%)	9,569 (35.4%)	**<0.001**
**Markers of acute organ failure**			
Sepsis	9,423 (6.3%)	4,042 (14.9%)	**<0.001**
Encephalitis	14 (0.01%)	20 (0.07%)	**<0.001**
Mild liver disease	1,267 (0.8%)	378 (1.4%)	**<0.001**
Severe liver disease	2,216 (1.5%)	1,923 (7.1%)	**<0.001**
Mechanical ventilation	2,720 (1.8%)	9,422 (34.8%)	**<0.001**
Extracorporeal membrane oxygenation (ECMO)	65 (0.04%)	1,389 (5.1%)	**<0.001**
Proteinuria	93 (0.1%)	42 (0.2%)	**<0.001**
Dialysis	1,522 (1.0%)	4,053 (15.0%)	**<0.001**
**Adverse events during hospitalization**			
In-hospital case-fatality	21,216 (14.2%)	10,391 (38.4%)	**<0.001**
MACCE	23,696 (15.9%)	11,328 (41.9%)	**<0.001**
Cardio-pulmonary resuscitation	1,099 (0.7%)	1,760 (6.5%)	**<0.001**
Venous thromboembolism (VTE)	2,992 (2.0%)	1,995 (7.4%)	**<0.001**
Acute kidney failure	12,144 (8.1%)	9,931 (36.7%)	**<0.001**
Myocarditis	126 (0.1%)	100 (0.4%)	**<0.001**
Myocardial infarction	1,624 (1.1%)	1,129 (4.2%)	**<0.001**
Stroke (ischemic or hemorrhagic)	2,206 (1.5%)	990 (3.7%)	**<0.001**
Intracerebral bleeding	279 (0.2%)	297 (1.1%)	**<0.001**
Gastro-intestinal bleeding	2,133 (1.4%)	815 (3.0%)	**<0.001**
Transfusion of blood constituents	5,906 (4.0%)	7,968 (29.5%)	**<0.001**

COVID-19, coronavirus disease 2019; ICU, intensive care unit; VTE, venous thrombo-embolism; MACCE, major adverse cardiovascular and cerebrovascular events; ARDS, acute respiratory distress syndrome.

*P*-values < 0.05 were considered to be statistically significant.

### Outcomes of COVID-19-patients admitted to ICU vs. those without ICU treatment

MACCE (41.9 vs. 15.9%, *P* < 0.001) and in-hospital case-fatality (38.4 vs. 14.2%, *P* < 0.001) rates were substantially higher in patients with COVID-19-infection treated in ICU than in those without ICU treatment (Table 1). ICU treatment was independently associated with increased in-hospital case-fatality rate [OR 5.49 (95% CI 5.30–5.68), *P* < 0.001].

**Figure 3:**
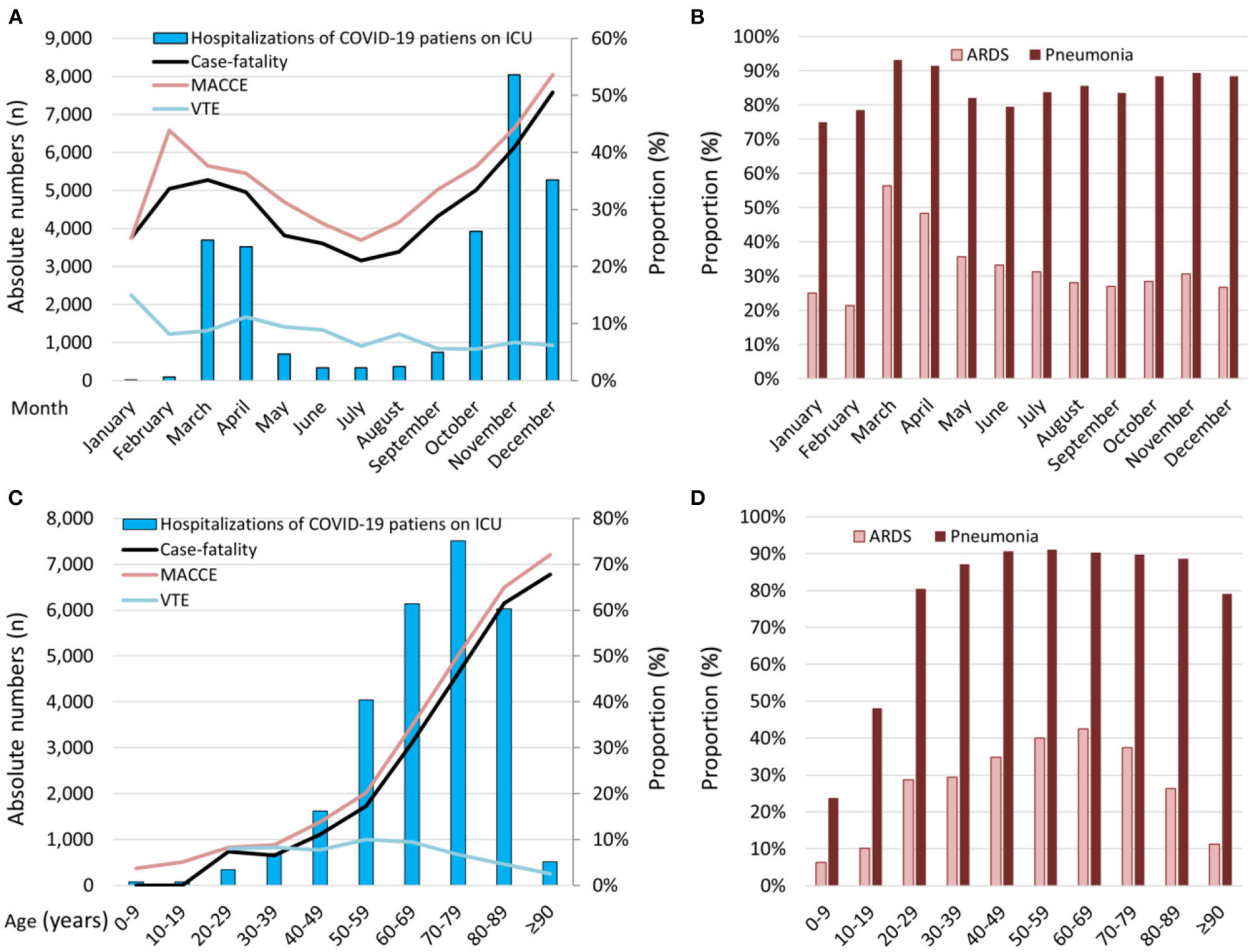
Temporal trends regarding total numbers of patients with COVID-19-infection admitted to ICU, in-hospital case-fatality, MACCE, and VTE rate. **(A)** Temporal trends regarding total numbers of patients with COVID-19-infection admitted to ICU (absolute numbers: blue bars) and rates of case-fatality, MACCE, and VTE stratified for months (lines). **(B)** Temporal trends regarding proportion of ARDS and pneumonia in patients with COVID-19-infection admitted to ICU stratified for months. **(C)** Temporal trends regarding total numbers of patients with COVID-19-infection admitted to ICU (absolute numbers: blue bars) and rates of case-fatality, MACCE, and VTE stratified for age decades (lines). **(D)** Temporal trends regarding proportion of ARDS and pneumonia in patients with COVID-19-infection admitted to ICU stratified for age decades. COVID-19, coronavirus disease 2019; ICU, intensive care unit; VTE, venous thromboembolism; MACCE, major adverse cardiovascular and cerebrovascular events; ARDS, acute respiratory distress syndrome.

It has to be pointed out that the in-hospital case-fatality rate of COVID-19 patients on ICU was highest in months with high numbers of ICU admissions of COVID-19 patients (Figure 3A). In addition, the case-fatality rate of COVID-19 patients treated on German ICUs increased substantially with patients age (Figure 3C). Highest proportion of ARDS cases were observed in the initial phase of the pandemic during spring 2020 (March-April) and in the 6st to 8th decade of patients' life (Figures 3B, D).

The rates of the following acute organ failures were increased in ICU patients: the rate of myocardial infarction was nearby 4-fold, whereas the rate of myocarditis was 3-fold increased and the stroke rate more than doubled in patients treated on ICU. While the rate of sepsis was more than doubled, occurrence of encephalitis was 7-fold increased and that of severe liver disease was nearby 5-fold inclined. Additionally, all investigated bleeding events and need for transfusion of blood constituents occurred significantly more often in ICU admitted patients (Table 1). Beside the bleeding events, the rate of venous thromboembolism was also more than 3-fold higher in ICU patients. Furthermore, the risk for acute kidney injury was more than 4-fold higher and, consequentially, dialysis was 15-fold more often performed in patients with COVID-19-infection treated on ICU (Table 1).

### Predictors of ICU admission of COVID-19-patients

Male sex [OR 1.96 (95% CI 1.90–2.01), *P* < 0.001] and age younger than 70 years [OR 1.47 (95% CI 1.43–1.52), *P* < 0.001] were independent risk factors of ICU admission (Table 2).

**Table 2:** Association between different conditions and ICU-treatment in COVID-19-patients (univariate and multivariate logistic regression model).

	**Univariate regression model**		**Multivariate regression model** ^*^	
	**OR (95% CI)**	***P*** **-value**	**OR (95% CI)**	***P*** **-value**
Age (per year)	1.003 (1.002–1.004)	**<0.001**	0.994 (0.993–0.995)	**<0.001**
Age ≥70 years	0.926 (0.903–0.951)	**<0.001**	0.681 (0.660–0.702)	**<0.001**
Female sex	0.504 (0.491–0.518)	**<0.001**	0.511 (0.497–0.526)	**<0.001**
**Cardiovascular risk factors**				
Obesity	2.536 (2.421–2.655)	**<0.001**	2.201 (2.097–2.310)	**<0.001**
Diabetes mellitus	1.769 (1.721–1.819)	**<0.001**	1.479 (1.436–1.524)	**<0.001**
Essential arterial hypertension	1.354 (1.319–1.390)	**<0.001**	1.276 (1.240–1.314)	**<0.001**
Hyperlipidemia	1.241 (1.200–1.284)	**<0.001**	0.908 (0.874–0.942)	**<0.001**
**Comorbidities**				
Coronary artery disease	1.594 (1.541–1.648)	**<0.001**	1.088 (1.048–1.130)	**<0.001**
Heart failure	2.021 (1.958–2.085)	**<0.001**	1.719 (1.658–1.783)	**<0.001**
Peripheral artery disease	1.583 (1.484–1.688)	**<0.001**	1.041 (0.972–1.114)	0.254
Atrial fibrillation/flutter	1.836 (1.783–1.891)	**<0.001**	1.566 (1.514–1.620)	**<0.001**
Chronic obstructive pulmonary disease	1.610 (1.539–1.684)	**<0.001**	1.263 (1.204–1.324)	**<0.001**
Chronic renal insufficiency (glomerular filtration rate <60 ml/min/1,73 m^2^)	1.238 (1.196–1.281)	**<0.001**	0.872 (0.839–0.906)	**<0.001**
Cancer	1.189 (1.124–1.257)	**<0.001**	1.175 (1.110–1.245)	**<0.001**
Charlson comorbidity index	1.146 (1.141–1.152)	**<0.001**	–	
**Respiratory manifestations of COVID-19**				
Pneumonia	6.609 (6.352–6.877)	**<0.001**	6.421 (6.164–6.689)	**<0.001**
Acute respiratory distress syndrome	39.746 (37.791–41.802)	**<0.001**	35.906 (34.104–37.803)	**<0.001**
**Adverse events during hospitalization**				
Cardio-pulmonary resuscitation	9.370 (8.680–10.115)	**<0.001**	7.431 (6.862–8.047)	**<0.001**
Venous thromboembolism	3.887 (3.668–4.120)	**<0.001**	3.782 (3.560–4.018)	**<0.001**
Acute kidney failure	6.540 (6.341–6.746)	**<0.001**	5.987 (5.790–6.191)	**<0.001**
Myocarditis	4.386 (3.372–5.705)	**<0.001**	3.744 (2.848–4.922)	**<0.001**
Myocardial infarction	3.954 (3.661–4.271)	**<0.001**	3.158 (2.908–3.429)	**<0.001**
Stroke (ischemic or hemorrhagic)	2.529 (2.344–2.729)	**<0.001**	2.277 (2.104–2.465)	**<0.001**
**Sepsis**	2.603 (2.503–2.708)	**<0.001**	2.529 (2.427–2.634)	**<0.001**
**Encephalitis**	7.878 (3.979–15.598)	**<0.001**	7.384 (3.652–14.929)	**<0.001**
**Mild liver disease**	1.653 (1.473–1.856)	**<0.001**	1.326 (1.176–1.495)	**<0.001**
**Severe liver disease**	5.072 (4.764–5.399)	**<0.001**	4.129 (3.868–4.408)	**<0.001**
Intracerebral bleeding	5.920 (5.025–6.975)	**<0.001**	5.485 (4.626–6.504)	**<0.001**
Gastro-intestinal bleeding	2.140 (1.972–2.322)	**<0.001**	1.907 (1.751–2.076)	**<0.001**
Transfusion of blood constituents	10.121 (9.755–10.502)	**<0.001**	10.131 (9.735–10.542)	**<0.001**

^*^Adjusted for age, sex, diabetes mellitus, cancer, heart failure, coronary artery disease, chronic obstructive pulmonary disease, essential arterial hypertension, chronic renal insufficiency (glomerular filtration rate <60 ml/min/1,73 m^2^), atrial fibrillation/flutter, hyperlipidemia, and obesity.

COVID-19, coronavirus disease 2019; ICU, intensive care unit; ARDS, acute respiratory distress syndrome.

*P*-values < 0.05 were considered to be statistically significant.

Regarding CVRF, obesity [OR 2.20 (95% CI 2.10–2.31), *P* < 0.001] as well as diabetes mellitus [OR 1.48 (95% CI 1.44–1.53), *P* < 0.001] were independent predictors of an increased need of ICU treatment (Table 2).

Interestingly, the association of atrial fibrillation/flutter as well as heart failure with ICU treatment were stronger than that of coronary artery disease and chronic obstructive pulmonary disease, which were also associated with ICU admission (Table 2).

The severe respiratory manifestations of COVID-19 pneumonia [OR 6.42 (95% CI 6.16–6.69), *P* < 0.001] and ARDS [OR 35.91 (95% CI 34.10–37.80), *P* < 0.001] were strongly and independently associated with ICU-admission. As expected, all adverse in-hospital events and acute organ failures were also associated with ICU-admission (Table 2).

### Risk factors for in-hospital death in COVID-19-patients treated on ICU

Increasing age, male sex, obesity, diabetes mellitus and the CVD heart failure, atrial fibrillation/flutter as well as peripheral artery disease, but also chronic obstructive pulmonary disease, chronic renal insufficiency (glomerular filtration rate <60 ml/min/1.73 m^2^) and cancer were independent risk factors for in-hospital death in COVID-19-patients treated on ICUs in Germany (Table 3). Aggravated respiratory manifestations of COVID-19-infection including pneumonia as well as ARDS were associated with more than 3-fold risk for in-hospital death in ICU-patients. In addition, as expected, adverse events during hospitalization were also accompanied by increased risk for in-hospital death (Table 3).

**Table 3:** Impact factors for in-hospital case-fatality in COVID-19-patients treated on ICU (univariate and multivariate logistic regression model).

	**Univariate regression model**		**Multivariate regression model** ^*^	
	**OR (95% CI)**	***P*** **-value**	**OR (95% CI)**	***P*** **-value**
Age (per year)	1.069 (1.067–1.072)	**<0.001**	1.068 (1.065–1.070)	**<0.001**
Age ≥70 years	4.071 (3.861–4.293)	**<0.001**	3.553 (3.352–3.765)	**<0.001**
Female sex	0.964 (0.915–1.015)	0.161	0.763 (0.720–0.809)	**<0.001**
**Cardiovascular risk factors**				
Obesity	0.814 (0.750–0.884)	**<0.001**	1.127 (1.028–1.236)	**<0.001**
Diabetes mellitus	1.291 (1.227–1.358)	**<0.001**	1.107 (1.045–1.172)	**0.001**
Essential arterial hypertension	0.965 (0.919–1.014)	0.161	0.711 (0.672–0.752)	**<0.001**
Hyperlipidemia	1.040 (0.976–1.108)	0.225	0.725 (0.674–0.779)	**<0.001**
**Comorbidities**				
Coronary artery disease	1.649 (1.553–1.751)	**<0.001**	0.997 (0.929–1.070)	0.937
Heart failure	2.037 (1.926–2.155)	**<0.001**	1.267 (1.187–1.352)	**<0.001**
Peripheral artery disease	1.952 (1.741–2.189)	**<0.001**	1.293 (1.141–1.465)	**<0.001**
Atrial fibrillation/flutter	2.283 (2.163–2.409)	**<0.001**	1.295 (1.219–1.376)	**<0.001**
Chronic obstructive pulmonary disease	1.577 (1.456–1.709)	**<0.001**	1.195 (1.095–1.303)	**<0.001**
Chronic renal insufficiency (glomerular filtration rate <60 ml/min/1,73 m^2^)	2.257 (2.119–2.403)	**<0.001**	1.337 (1.245–1.435)	**<0.001**
Cancer	1.738 (1.570–1.924)	**<0.001**	1.697 (1.520–1.895)	**<0.001**
Charlson comorbidity index	1.410 (1.394–1.425)	**<0.001**	–	
**Respiratory manifestations of COVID-19**				
Pneumonia	2.725 (2.480–2.994)	**<0.001**	3.441 (3.104–3.815)	**<0.001**
Acute respiratory distress syndrome	2.106 (2.001–2.217)	**<0.001**	3.049 (2.872–3.237)	**<0.001**
**Adverse events during hospitalization**				
Cardio-pulmonary resuscitation	6.164 (5.497–6.913)	**<0.001**	7.130 (6.291–8.082)	**<0.001**
Venous thromboembolism	1.075 (0.979–1.180)	0.129	1.367 (1.235–1.513)	**<0.001**
Acute kidney failure	4.305 (4.084–4.538)	**<0.001**	3.972 (3.747–4.211)	**<0.001**
Myocarditis	0.686 (0.447–1.053)	0.085	0.921 (0.562–1.507)	0.743
Myocardial infarction	1.383 (1.227–1.559)	**<0.001**	1.002 (0.876–1.145)	0.979
Stroke (ischemic or hemorrhagic)	1.755 (1.545–1.993)	**<0.001**	1.851 (1.611–2.127)	**<0.001**
Intracerebral bleeding	2.679 (2.116–3.391)	**<0.001**	4.676 (3.620–6.041)	**<0.001**
Gastro-intestinal bleeding	1.825 (1.587–2.099)	**<0.001**	1.543 (1.325–1.797)	**<0.001**
Transfusion of blood constituents	2.321 (2.201–2.449)	**<0.001**	2.339 (2.203–2.482)	**<0.001**

^*^Adjusted for age, sex, diabetes mellitus, cancer, heart failure, coronary artery disease, chronic obstructive pulmonary disease, essential arterial hypertension, chronic renal insufficiency (glomerular filtration rate <60 ml/min/1,73 m^2^), atrial fibrillation/flutter, hyperlipidemia, and obesity.

COVID-19, coronavirus disease 2019; ICU, intensive care unit; ARDS, acute respiratory distress syndrome.

*P*-values < 0.05 were considered to be statistically significant.

## Discussion

One of the most critical determinants in the worldwide health care management of the COVID-19 pandemic are the local ICU capacities (3, 8, 9). This was impressively obvious in several epicenters of the COVID-19 pandemic with dramatic high case-fatality rates due to overloaded local health care and especially ICU capacities (3, 10, 11, 24–26).

Our study analyzing more than 175,000 hospitalizations of inpatients with COVID-19 infection revealed a substantially higher in-hospital case-fatality rate (24.2% higher) and MACCE rate (26.0% higher), if an ICU treatment was required in the not-vaccinated German population. As the vaccination program started in Germany not before late December 2020, the wide majority of hospitalized patients with COVID-19 during the year 2020 were not vaccinated and vaccination has no influence on the outcomes of our present study. The need for ICU-treatment was independently associated with 5.4-fold elevated risk for in-hospital case-fatality rate. In accordance with our results, previously published studies have also revealed high case-fatality rates of COVID-19-patients admitted to ICUs and emphasized the importance of accessible ICU beds, ventilator capacities and trained staff to manage the COVID-19 pandemic adequately (3, 8, 27). This was underlined by data of the United States of America showing that 79% of the hospital beds at the ICUs were occupied by COVID-19-patients at the peak of the pandemic during January 2021 (28). The proportion of patients with COVID-19-infection, who were transferred to an ICU in Germany, was 15.4% and therefore, comparable to proportions in France (16.4%) (29), United Kingdom (17.0%) (30), and in the Unites States of America (10.2–19.6%) (31–33), but lower than in other countries such as Spain (26.3%) (34) or Iran (19.0%) (35). Pooled ICU admission rate among 17,639 hospitalized COVID-19 patients meta-analyzed from eight studies worldwide was reported as 21% (36). While the highest absolute numbers of total ICU admissions due to COVID-19-patients in the year 2020 were observed in spring and winter, the monthly percentage of COVID-19 patients treated at the ICU of German hospitals decreased over time from 32.8% in January 2020 to a minimum of 14.8% at November 2020 and revealed only small variations between May and December 2020.

In accordance with our finding of a high case-fatality rate of COVID-19-patients treated in German ICUs during spring and winter of the year 2020 when ICU demands were highest (Figure 3A), other studies have also shown that the ICU capacities and the ICU demand are important factors for COVID-19 patients' outcome (33, 37). COVID-19 patients who needed ICU-treatment during periods of increased COVID-19 ICU demand had an increased risk of mortality compared with patients treated during periods of low COVID-19 ICU demand, whereas no association between COVID-19 ICU demand and mortality was observed for patients with COVID-19 treated outside the ICUs (37). This finding is of outstanding interest for adequate pandemic management.

In addition, significant variations regarding in-hospital case-fatality rate across European countries were observed (38, 39). The in-hospital case-fatality rate of COVID-19 patients treated on ICU during the year 2020 in Germany identified by our study was 38.4% and therefore higher than the rates reported in studies from Spain, Andorra and Ireland (30.7%) (27), France (31.0%) (40), United Kingdom (32.0%) (30), Spain (16.7–34.0%) (41, 42), Netherlands (23.4–32.0%) (43), United States of America (21.0–29.7%) (32, 44), Sweden [17.4% (in-hospital mortality)−32.1% (60-day mortality after ICU discharge)] (45, 46), Iceland (14.8%) (47), was similar to rates in China (37.0%) (48) as well as in Denmark (37.0%) (49) and lower than in Iran (42.0%) (35), Russia (65.4%) (50), and Brazil (59.0%) (51). Two large review article including data of ICUs around the world reported summarized worldwide ICU mortality rates of 28.3% (36) and 35.5% (52), the second close to the value we calculated for Germany.

As aforementioned, inter-country differences regarding the COVID-19 patients' outcome are strongly impacted by ratio of ICU capacities and ICU demand (33, 37). In addition, patients' age, sex-distribution and comorbidities are important for these observed differences (27, 31, 43, 46, 53). In particular, the age-dependency of COVID-19 case-fatality is well-known and very important in this context (3). Therefore, variations in median age of the different COVID-19-cohorts in the different countries influence the case-fatality rates and might contribute to these variations. For example, in the Swedish COVID-19 intensive care cohort (46) as well as in cohort studies of the United States of America (31), the median age of the ICU patients was more than 10 years lower (31, 46) and in the cohort study in Spain, Andorra and Ireland the median age was 8 years lower than in Germany (27). In line with this age-comparison, age ≥70 years was a strong predictor of in-hospital death of COVID-19-patients admitted to ICUs in Germany. In addition, aggravated respiratory status such as pneumonia and ARDS as well as acute kidney injury were strongly and independently associated with increased in-hospital case-fatality. However, since not all COVID-19 patients without dyspnoea, who were treated on normal ward, will be and were examined with X-ray, the proportion of pneumonia in this patient group might be underestimated.

The prevalence rates of all investigated acute organ failures were substantially higher in ICU patients than in the COVID-19 patients treated on normal ward. Especially, rates of cardiac involvement, but also stroke, encephalitis and sepsis were substantially elevated. Sepsis is a common complication in COVID-19 patients and was detected in 7.6% of all hospitalized COVID-19 patients and in more than 14% of the COVID-19 patients treated on ICU in Germany in the year 2020. However, studies indicate that this number might be underestimated and the real rate of viral sepsis in hospitalized COVID-19 patients might be significantly higher (54). Cardiac involvement is a known phenomenon and complication in patients suffering from COVID-19-infection (3, 55–58) and comprises predominantly myocardial infarction as well as myocarditis. COVID-19 was identified as a risk factor for acute myocardial infarction and myocarditis (3, 55–60). In studies, SARS-CoV-2 was associated with an increased risk of both arterial and venous thrombotic complications and in particular the risk of myocardial infarction was approximately doubled in the first 7 days after COVID-19 diagnosis (60). Myocarditis incidence of hospitalized patients was reported ranging between 2.4 and 4.1 cases per 1,000 COVID-19 patients in a multi-center study of centers of different European countries and the United States of America (56, 58). Cerebral complications such as stroke and encephalitis were reported in studies (59, 61), but our data underlines the importance and impact of these widely overlooked complications. These different acute organ failures are key drivers of in-hospital mortality and therefore, the early detection of impending complications as well as prevention and treatment of these acute organ failures is of major interest for adequate management of COVID-19 patients. It is additionally of outstanding interest, that all investigated bleeding events occurred more often in COVID-19 patients admitted to ICUs. Bleeding events during hospitalization were in different studies strongly associated with increased in-hospital death (62, 63).

Although the COVID-19 pandemic was primarily managed by vaccination programme after the year 2020 (vaccination program started in Germany at late December 2020 and accelerated in the following years) (64), nevertheless, ~1/4 of the German population has still no basic immunization by a COVID-19 vaccination at the beginning of the year 2023 (65). Therefore, risk factors for ICU admission in not-vaccinated patients are still important for health care management. While the understanding of impacting factors on ICU admission and outcome is crucial for adequate health care planning, decision making, and pandemic management (3, 6, 66), the understanding of these factors remains still unsatisfying (66). We identified male sex as well as obesity and diabetes mellitus as independent predictors for an increased probability of ICU treatment. These findings is consistent with previously published study results in which obesity and diabetes mellitus were associated with aggravated outcome in hospitalized patients with COVID-19 (3, 7, 67–70).

In accordance with contemporary literature (71), the prevalence of CVD is distinctly higher in COVID-19-patients requiring ICU care. Interestingly, the association of atrial fibrillation/flutter as well as heart failure with ICU-admission were stronger than the associations of coronary artery disease and chronic obstructive pulmonary disease with ICU-admission, respectively. Atrial fibrillation is the most common arrhythmia in patients with COVID-19-pneumonia affecting ~20% of the patients with severe COVID-19 pneumonia during ICU stay (72). In patients with COVID-19, arrhythmias and chronic heart failure are key factors of the development of acute heart failure (73) and heart failure diagnosis is associated with aggravated mortality in COVID-19 patients (3, 73). COVID-19-infection is associated with increased risk of arterial und venous thrombosis with resulting ischemic events and patients with myocardial infarction infected by COVID-19 had an unfavorable outcome in comparison to those patients without COVID-19 infection (3, 60, 71, 74–77). Among COVID-19 patients, higher proportions of patients with COPD have to be admitted to ICU and treated with mechanical ventilation (78). Consequently, COPD is an independent risk factor for ICU admission and all-cause mortality in COVID-19 patients (78, 79).

In line with our results, other studies identified patients age, arterial hypertension, diabetes mellitus, chronic renal failure, bronchial asthma, obesity and immunosuppression as independently associated with ICU admission during COVID-19-infection (46, 80). Nevertheless, it has to be mentioned, that we were not able to distinguish whether the adverse in-hospital events occurred during, before or after ICU treatment during the hospitalization of the COVID-19 patients. However, the aim of the study was to emphasize and illustrate the stress and strain of the ICU in Germany.

Total number of patients necessitating ICU admission in relation to provided ICU capacities have to be taken into account for adequate health care and pandemic planning (3, 8, 9). Based on the knowledge regarding regional differences, epicenters of the COVID-19 pandemic with very high mortality rates, it is of outmost importance to identify trends and factors affecting ICU admission to avoid a critical overload of the healthcare system and particularly of the ICUs with increasing mortality rates (5, 8, 9).

## Limitations

Certain limitations of the present study merit consideration: First, as our results are based on administrative data, we cannot exclude misclassification or inconsistencies. Additionally, our analysis of the German nationwide inpatient sample was not pre-specified and thus, findings of the study can only be considered to be hypothesis-generating. Second, patients with confirmed COVID-19 infection, who died out of hospital, were not included in the German nationwide inpatient sample. Third, the German nationwide inpatient sample does not report follow-up-outcomes after the discharge from hospital. Fourth, coding on medical treatments is only incompletely captured (especially regarding immunotherapy such as dexamethasone, tocilizumab, anakinra, and baricitinib).

## Conclusion

During the year 2020, 15.4% of the hospitalized COVID-19-patients were admitted to ICUs in German hospitals. Important and independent risk factors for ICU admission are male sex, CVRF such as obesity and diabetes mellitus as well as several cardio-pulmonary diseases including atrial fibrillation/flutter, heart failure, coronary artery disease and chronic obstructive pulmonary disease. ICU-admission was accompanied by a case-fatality rate of 38.4% and for this substantially higher than the rate 14.2% on normal-ward treatment. COVID-19 patients who were treated in ICUs during periods of increased ICU demand had an increased risk of mortality compared to patients treated during periods of low COVID-19 ICU demand. These findings highlight to draw more attention to predictors for ICU admission in patients hospitalized with COVID-19 in order to optimize monitoring and prevention strategies, avoid critical overload of the healthcare system and particularly of the ICUs in order to prevent the subsequent increase in mortality rates.

## Data availability statement

The raw data supporting the conclusions of this article will be made available by the authors, without undue reservation.

## Ethics statement

Ethical review and approval was not required for the study on human participants in accordance with the local legislation and institutional requirements. Written informed consent from the participants' legal guardian/next of kin was not required to participate in this study in accordance with the national legislation and the institutional requirements.

## Author contributions

KK and LH conceived this study, led the writing of the paper, accessed and verified the data, and contributed to the study design. LH, IF, LV, SKoe, JW, SB, FS, CE-K, SKon, TM, and IS commented on the paper, oversaw the analysis, and edited the final manuscript. KK led the data analysis with support from LH. All authors had full access to all the data, contributed to drafting the paper, revised the manuscript for important intellectual content, and had final responsibility for the decision to submit for publication.
